# COVID-19: A review and considerations for the resumption of activities in an IVF laboratory and clinic in Brazil

**DOI:** 10.5935/1518-0557.20200102

**Published:** 2021

**Authors:** Ianaê Ceschin, Taccyanna Ali, Cristina Carvalho, Mariane Uehara, Priscila Motta, Marcia Riboldi

**Affiliations:** 1 Feliccità Instituto de Fertilidade - Curitiba, Paraná, Brasil; 2 Laboratório Igenomix - Laboratório de Genética e Medicina Reprodutiva - São Paulo, São Paulo, Brasil; 3 Centro de Estudos sobre o Genoma Humano e Células-Tronco (CEGH-CEL), Departamento de Biologia Evolutiva, Instituto de Biociências - Universidade de São Paulo, São Paulo, Brasil

**Keywords:** COVID-19, SARS-CoV-2, pandemic, IVF laboratory, human reproduction

## Abstract

COVID-19 has caused radical effects on the daily lives of millions of people. The causal agent of the current pandemic is SARS-CoV-2, a virus that causes symptoms related to the respiratory system, leading to severe complications. In the *in vitro* fertilization (IVF) universe, there are several protocols for infection control and laboratory safety. Some professional associations have issued guidelines recommending measures involving patient flow and IVF practices. This study presents a review and considerations for the resumption of activities in IVF laboratories and clinics in Brazil during the COVID-19 pandemic, according to the guidelines and statements from professional organizations and societies in reproductive medicine.

## 1. Background

The COVID-19 pandemic has caused radical effects on the daily lives of millions of people. It emerged in December 2019 in Wuhan, China (Huang *et al*., 2020). On January 30, 2020, the World Health Organization (WHO) declared it a global health emergency. Due to the increase in the number of infected people and deaths, on March 11, the WHO (2020a) declared COVID-19 a pandemic. At the time of this writing (August 08, 2020), Brazil has become the second country, after the US, to register more than 100,000 deaths from this disease (Secretaria Estaduais de Saúde - Brasil, 2020). Figure 01 shows the total number of COVID-19 deaths on a linear scale in Brazil.

**Figure 1:**
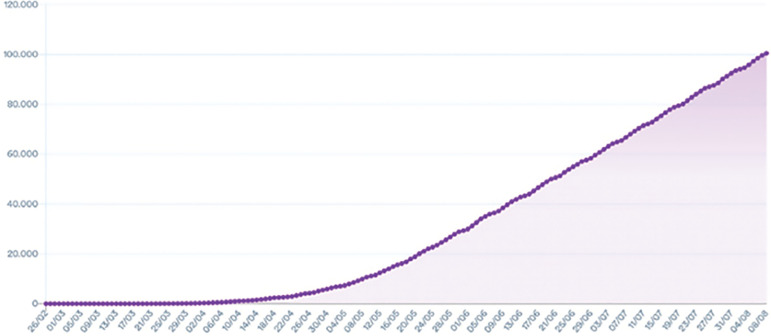
Graph showing the total of deaths due to COVID-19 in Brazil (Secretarias Estaduais de Saúde - Brasil, 2020).

COVID-19 is an infectious disease caused by the new coronavirus associated with severe acute respiratory syndrome (SARS-CoV-2) (Huang *et al*., 2020). The spread among people occurs through respiratory droplets emitted by an infected individual through coughing or sneezing, subsequently inhaled by a healthy person nearby (Fiorillo *et al*., 2020). The most common symptoms for the onset of the disease are fever (40.9%), cough (31.7%), fatigue or myalgia (18.4%), sputum production (11.2%), and headache (3.8%) (Huang *et al*., 2020). A study reported maternal deaths (Hantoushzadeh *et al*., 2020), one detected the pathogen in placenta specimens of a second-trimester miscarriage (Baud *et al*., 2020), and other found SARS CoV-2 in semen samples (Li *et al*., 2020a), but the effects on pregnancy are still under debate (Gao *et al*., 2020).

SARS-CoV-2 shows approximately 79% genetic similarity to SARS-CoV and ~50% similarity to Middle East respiratory syndrome coronavirus (MERS-CoV) (Lai *et al*., 2020; Zu *et al*., 2019). SARS-CoV-1 caused an outbreak in 2002 with Severe Acute Respiratory Syndrome (SARS) (Ksiazek *et al*., 2003), and MERS-CoV was the agent responsible for the Eastern Respiratory Syndrome Medium (MERS) in 2012 (Zaki *et al*., 2012). Although the lethality rate of COVID-19 has been significantly lower than that of the SARS and MERS epidemics, the SARS-CoV-2 virus transmission is much larger than that of the previous viruses, with a much higher total number of deaths (Table 01). The preventive measures are the current strategy to limit the spread of cases (Cascella *et al*., 2020).

**Table 1:** COVID-19 and previous pandemics caused by coronaviruses (Wu & McGoogan, 2020; WHO, 2003; 2019; 2020b).

Disease	COVID-19	SARS	MERS
Virus	Betacoronavirus SARS-COV-2	Betacoronavirus SARS-COV-1	Betacoronavirus MERS-COV
Origin	Whuan, China	Guandong, China	Saudi Arabia
Onset	December 2019 - Currently	2002 - July 2003	June 2012 -still active
Spread	194 countries	29 countries	27 countries
Cases	20,162,474 reported	8,422 reported	2,494 reported
Deaths	737,417	916	858
Reproductive no.	≈3	≈2-3	≈1
Age (range)	59 (10-89) years	40 (1-91) years	50 (1-94) years
Sex Ratio (M:F)	56:44	43:57	64.5:35.5
Mortality	Currently estimated 2.3%	9.6%	35-40%

According to these facts, many human reproduction societies issued recommendations on how to deal with the pandemic and *in vitro* fertilization (IVF) procedures. This study presents a review and considerations for the resumption of activities of IVF laboratories and clinics in Brazil during the COVID-19 pandemic era, according to the guidelines and statements from professional organizations and societies in reproductive medicine.

## 2. Fertility treatments

The American Society for Reproductive Medicine (ASRM) and the European Society for Reproduction and Embryology (ESHRE) have recommended cancelling fertility treatments, except in poor responders, who can still undergo treatment, although this may lead to additional stress for couples who badly want to have a child (ESHRE, 2020; ASRM, 2020; Anifandis *et al*., 2020). These societies have recommended suspending the onset of new treatments and an alternative freeze-all protocol in cases where couples have already undergone human chorionic gonadotrophin triggering (ESHRE, 2020; ASRM, 2020; Anifandis *et al*., 2020).

The Brazilian Society for Assisted Human Reproduction (SBRA), the Brazilian Society for Human Reproduction (SBRH), and the Latin American Assisted Reproduction Network (REDLARA) have stated that individual cases must be discussed with a physician, because there are unique situations, in which postponing the treatment could reduce the likelihood of successful pregnancy (SBRH, 2020; SBRA & REDLARA, 2020). On June 08, 2020, the SBRA, the SBRH, PRONUCLEO, and other societies from Latin America related to the field of reproductive medicine have allowed the resumption of operations in fertility clinics. They have recommended prioritizing the individual cases with a well-documented medical record (SBRA et al., 2020).

## 3. Fertility clinic and internal policies

As reported by the WHO, health facilities have to implement internal policies to ensure that all necessary preventive and protective measures are taken to minimize occupational safety and health risks. The clinical director or managers must provide information, instruction, and training on Occupational Safety and Health (OSH). The training includes information on how to wear Personal Protective Equipment (PPE), and the handling and disposal of infectious waste (WHO, 2020c). The establishment must provide and keep control of Collective Protection Equipment (CPE) and cleaning products to protect the worker during patient care (ANVISA, 2020). The IVF clinic must manage the risks involved during patient-care and create respective prevention and control measures (ANVISA, 2020).

### 3.1. Physical barriers

Coronavirus spreads primarily through droplets generated by infected individuals during coughing, sneezing, or speaking (Allam *et al*., 2020). Environmental surfaces are more likely to be contaminated with the COVID-19 virus in healthcare settings where specific medical procedures are carried out (Ye *et al*., 2020; Ong *et al*., 2020; Faridi *et al*., 2020). Ong *et al*. (2020) evaluated the presence of coronavirus in a hospital room of COVID-19 patients. Some surfaces, such as the toilet bowl and the sink, were positive. Room air samples and samples collected after cleaning were negative. Due to the potential of the virus to survive in the environment for several days, facilities and areas potentially contaminated with SARS-CoV-2 must be cleaned before being reused, with products containing antimicrobial agents known to be effective against coronaviruses (ECDPC, 2020; Oliveira *et al*., 2016). Specific COVID-19 sanitation procedures should be implemented in the case of COVID-19 positive patients or staff members (ESHRE, 2020). All medical staff should remain diligent about strictly following the recommended PPE guidelines and ensure both availability and utilization of PPE for themselves, their healthcare team, and their patients (ASRM, 2020). In Tables 02 and 03, we show other measures to avoid the spread of COVID-19 cases.

**Table 2:** Suggestions to limit the spread of COVID-107 19 for the clinic staff and patients.

Sector	Measures	PPE required
Valet parking	-Ensure alcohol-based hand rub is available. -Provide a moistened cloth with 70% alcohol solution to clean steering wheels and car doors.	-Surgical or fabric mask
Office administration	-Ensure alcohol-based hand rub is available. -Ensure proper ventilation with outside air. -Encourage employees to maintain at least one meter from other employees when possible. -Encourage flexible work hours or rotational shifts. -Review cleaning and disinfection procedures for furniture such as desks, armchairs, and other personal use objects such as computers, pens, etc.	-Surgical or fabric mask
Waiting area- patient reception area	-Encourage employees to maintain at least one meter from other employees when possible. -Install physical barriers in reception areas to limit contact between staff and patients. -Limit the use of shared items by patients (e.g., pens, clipboards, phones). -Encourage respiratory hygiene/cough etiquette. -Provide disposable tissue and foot-operated waste bin in the waiting room. Ensure alcohol-based hand rub is available and encourage hand hygiene (e.g., hand washing with non-antimicrobial soap and water, alcohol-based hand rub, or antiseptic hand wash) after having contact with respiratory secretions and contaminated objects/materials -Ensure proper ventilation with outside air. -Review the cleaning and the disinfection procedures for furniture such as desks, armchairs, for example. Chairs or armchairs and benches must be made of washable material or easy to clean.	-Receptionist or Secretary: Surgical mask -Patients with or without clinical symptoms of respiratory disease: surgical mask.
Cleaning staff/waitress	-Ensure alcohol-based hand rub is available -Clean frequently touched surfaces. -Cleaning staff should not serve food and drinks to patients	-Cleaning staff N95 mask, disposable apron, goggles or face shield and gloves. -Waitress: surgical mask or tissue, cap and gloves

**Table 3:** Suggestions to limit the spread of COVID-19 for the clinic staff and patients where clinical procedures are performed (WHO, 2020c; ANVISA, 2020).

Sector	Measures	PPE required
Consultation rooms	-Avoid accompanying persons -Encourage physicians to maintain at least one meter from patients when possible -Review cleaning and disinfection procedures for furniture such as desks, armchairs, and other objects of personal use such computers, pens, etc. -Ensure alcohol-based hand rub is available. -The cleaning employee must clean the workplace, such as desks, computers, and phones, before the start and after the workday	-Staff: surgical mask, apron, goggles or face shield, and depending on the procedure, gloves -Patients with or without clinical symptoms of respiratory disease; surgical mask
Ultrasound room	-Avoid accompanying persons -Ensure alcohol-based hand rub is available. -Review cleaning and disinfection procedures	-Staff: surgical mask, apron, goggles or face shield, and depending on the procedure, gloves -Patients without clinical symptoms of respiratory disease: surgical mask. Patients with clinical symptoms of respiratory disease: surgical mask, disposable apron, and disposable shoe covers *Staff should wear the N95 or PFF2 mask if the patient has clinical symptoms of respiratory disease.
Specimen collection (Blood/semen)	-Avoid accompanying persons -Ensure alcohol-based hand rub is available -Review cleaning and disinfection procedures	-Staff: surgical mask, apron, goggles or face shield, and depending on the procedure, gloves -Patients without clinical symptoms of respiratory disease: surgical mask. Patients with clinical symptoms of respiratory disease: surgical mask, disposable apron, and disposable shoe covers *Staff should wear the N95 or PFF2 mask if the patient has clinical symptoms of respiratory disease.
Room for oocyte retrieval/ testicular puncture	-Avoid accompanying persons -Ensure alcohol-based hand rub is available -Review cleaning and disinfection procedures	-Staff should wear the N95 or PFF2 mask if the patient has clinical symptoms of respiratory disease. -Patients: surgical mask, disposable apron, and disposable shoe covers

### 3.2 Staff and patient flow

Primary transmission is believed to occur through respiratory droplets from coughing and sneezing, and contagion requires proximity (less than 6 feet distance) between individuals (Cascella *et al*., 2020). One should strive to implement procedures for staff to work remotely or from home, understanding that such arrangements need to be individualized (ASRM, 2020). Subdivision of staff into mini teams reduces unnecessary exposure of patients and staff members, and encourages the cross-training of staff in the event of staff absences (ESHRE, 2020; ASRM, 2020).

Regarding patient flow, ASRM has recommended limiting the number of visitors to a single support person or encouraging alternative participation methods, such as by phone or video. When urgent procedures must be performed, minimize the time the patient is waiting in the reception area or waiting room (ASRM, 2020). PRONUCLEO issued a guideline with detailed recommendations on patient flow. Below, we show the suggestions according to ASRM, ESHRE and PRONUCLEO:

Before the consultation:

- When scheduling procedures, receptionists must provide patients with information: masks are necessary and avoid accompanying persons.
- Instruct patients with respiratory infection symptoms (fever, cough, runny nose, difficulty breathing) to postpone the procedure. However, if postponing the appointment is impossible, instruct them to report respiratory infection symptoms as soon as they arrive at the clinic.
- The procedures must be scheduled at intervals that allow the cleaning of the rooms.

During the consultation:

- The patients must complete the Health Status Questionnaire (QSS) daily.
- Minimize contact with the patient to what is strictly necessary during the procedure, always maintaining a distance of at least 1 meter.
- For so-called asymptomatic patients, we recommended the patient should wear a mask.
- In cases of symptomatic patients, inform the entire team involved in the procedure in advance.
- During the patient’s presence at the service, whether characterized as asymptomatic or proven to be infected, avoid sharing items such as pens, clipboards, computers, phones, and others. Establish a hygiene protocol between procedures with the appropriate teams involved.
- If possible and, according to ANVISA rules, obtain consent documents electronically to minimize the patient’s time in the unit where the procedure is performed.

#### 3.2.1 *Telemedicine*

According to Tuckson *et al*. (2017) telehealth, a term used to telemedicine, has been defined to exchange medical information from one site to another through electronic communication to improve a patient’s health. There are many digital platforms for this interaction between patients and physicians (Souza *et al*., 2020).

Telemedicine should be used for all treatment steps that do not require the physical presence of patients at the clinic, to reduce unnecessary visits and staff-patient contact (ESHRE, 2020). ASRM has encouraged the use of telemedicine for new and returning patients. These communication models could also be applied for planning treatments, mental health consultations, nurse counseling, and administrative discussions (ASRM, 2020). SBRA has also supported telemedicine use to perform patient consultations whenever possible in the meantime, to finish the reproductive treatment, a visit or a face-to-face consultation may be necessary.

## 4. Testing

On 18 March 2020, ANVISA established extraordinary and temporary rules to speed up the evaluation of new products by prioritizing the testing needed to detect the new coronavirus. This measure is part of the strategic actions to make products that can be used to face the COVID-19 pandemic more quickly, and maintain the safety and effectiveness criteria. Since the entire genetic sequence of the SARS-CoV-2 virus was uploaded to the Global Initiative on Sharing All Influenza Data (GISAID) platform on 10 January 2020, companies and research groups have developed many diagnostic kits for COVID-19. The availability of sequence data has facilitated the design of primers and probes needed for the development of SARS-CoV-2-specific testing (Tan, 2020). In Brazil, tests for COVID-19 consist of mostly immunoassays to detect antibodies or antigens, and molecular biology assays to detect RNA from the virus.

### 4.1 Immunoassays: Antibody or antigen detection

#### 4.1.1 *Point-Of-Care (POC) tests*

This kind of laboratory test is designed to be used directly at the site of patient care, which may comprise physicians’ offices, outpatient clinics, intensive-care units, emergency rooms, hospital laboratories, and even patients’ homes (Price, 2001). Their execution and results interpretation are carried out in a maximum of 30 minutes. The samples required are easily obtained, such as urine, blood, saliva, or nasopharyngeal swabs (Foo *et al*., 2009; Fox *et al*., 2011). In the setting of infectious diseases, most existing Point-Of-Care (POC) tests consist of immunoassays, namely agglutination, immunochromatographic, and immunofixation tests (Von Lode, 2005). Currently, more than half of the registrations granted by ANVISA for COVID-19 diagnosis consists of immunochromatographic tests for Immunoglobulin M (IgM) and Immunoglobulin G (IgG), and only a handful are approved for antigen detection (ANVISA, 2020).

Regarding the tests approved for antibody detection, all of them require a blood sample. A small sample of the patient’s blood is taken from a vein or a finger-prick device and dropped onto a spongy pad within the testing device. Few drops of a buffer are added to help the blood sample flow across the device. As the sample moves through the device, antibodies against SARS-CoV-2 present in the sample will attach to chemicals in the device, capturing the antibodies on the test and the control lines. This capturing and binding process results in a color change along the test and control lines, producing one, two, or three lines, depending on the type of antibodies present (IgM or IgG). Figure 02 shows the possible results with these assays. The appearance of a line for IgG or IgM indicates a positive test - showing the patient has been exposed to SARS-CoV-2. A control line must appear to show the assay has worked correctly.

**Figure 2:**
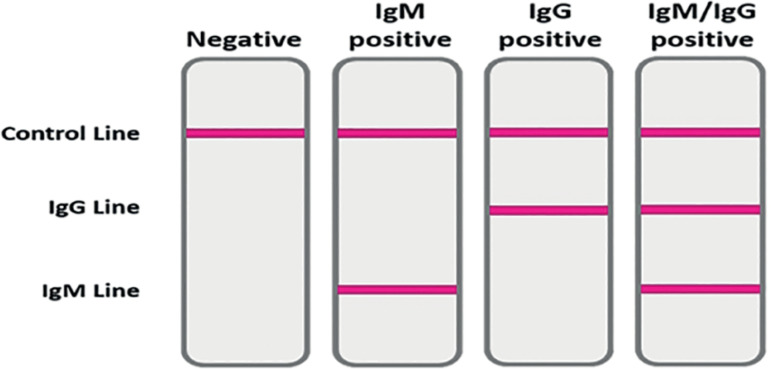
Possible results with POC tests.

#### 4.1.2 *Enzyme-Linked 206 Immunosorbent Assay (ELISA)*

An Enzyme-Linked Immunosorbent Assay (ELISA) is a microwell, plate-based assay technique designed to detect and quantify substances such as peptides, proteins, antibodies, and hormones. They can be qualitative or quantitative, and it takes 1-5 hours to get the results (Carter *et al*., 2020). In Brazil, kits available for ELISA can detect the Immunoglobulin A (IgA), Immunoglobulin M (IgM), or Immunoglobulin G (IgG). The plate wells are typically coated with a viral protein. If present, antiviral antibodies in the patient samples will bind to these proteins, and the bound antibody-protein complex can be detected with an additional tracer antibody to produce a colorimetric or fluorescent-based readout (Carter *et al*., 2020). The combination of IgM and IgG antibodies in the detection for COVID-19 disease proved to be the best sensitivity technique when compared with other isolated tests for each antibody (Li *et al*., 2020b).

#### 4.1.3 *Chemiluminescence immunoassay (CLIA)*

Regarding the diagnosis of COVID-19, the Chemiluminescence Immunoassay (CLIA) also detects IgG and IgM antibodies. The DZ-Lite SARS-CoV-2 test was developed by Diazyme, USA, which, based on the chemiluminescent assay principle, can analyze 50 samples in one hour. Another company, Snibe, China, with the MAGLUMIA CLIA test based on the same principle, can detect antibodies in a patient sample in 30 minutes. The automated chemiluminescence test can detect the antibodies generated against the pathogen, and, compared to the POC test; this methodology has a very high yield of samples that can be analyzed, and the ability to perform other tests (Cinquanta *et al*., 2017).

### 4.2 Molecular assays: RNA detection

#### 4.2.1 *Real-Time Reverse Transcriptase-PCR (RT-PCR)*

Along with advancements in medical diagnosis, nucleic acid detection-based approaches have become a rapid and reliable technology for viral detection. Among nucleic acid tests, Real-Time Reverse Transcriptase-PCR (RT-PCR) is of great interest today for SARS CoV-2 detection due to its benefits as a specific and easy qualitative assay (Corman *et al*., 2020). The RT-PCR starts with the conversion of viral genomic RNA into DNA by RNA-dependent DNA polymerase (reverse transcriptase). This reaction relies on small DNA- sequence primers designed to accurately recognize complementary sequences on the RNA viral genome and the reverse transcriptase to generate a short complementary DNA copy (cDNA) of the viral RNA (VanGuilder *et al*., 2008). For SARS-CoV-2 detection, a variety of RNA-gene targets are used by different manufacturers, with most tests targeting 240 or more of the Envelope (Env), Nucleocapsid (N), Spike (S), RNA-dependent RNA polymerase (RdRp), and ORF1 genes (Sethuraman *et al*., 2020).

In RT-PCR, DNA amplification is monitored in real-time as the PCR reaction progresses, because the reaction happens using a fluorescent dye or a sequence-specific DNA probe labelled with a fluorescent molecule and a quencher molecule, as in the case of TaqMan assays. An automated system then repeats the amplification process for about 40 cycles until the viral cDNA can be detected, usually by fluorescent or electrical signal (VanGuilder *et al*., 2008).

### 4.3 Who to test and the best technique to detect COVID-19

According to many societies, tests, whether by PCR or IgG / IgM / IgA antibodies, in patients or staff, will depend on local availability (SBRA *et al*., 2020; ESHRE, 2020). ASRM incorporates testing as part of patient and staff management strategies when these are accurate and available, it is recommended according to the last update on March 11, 2020. Testing could be used to guide patient management and inform the use of appropriate PPE to protect patients and staff against infection (ASRM, 2020). The WHO (2020d) recommends all employees working in healthcare organizations should be tested if possible.

Nucleic acid testing is the first method for diagnosing COVID-19 (CDC, 2020). However, if an asymptomatic patient was infected by SARS-CoV-2 but has recovered, only RT-PCR would not identify this prior infection, and control measures would not be enforced (Udugama *et al*., 2020), thus testing of paired serum samples with the initial RT-PCR can increase diagnostic accuracy (Sethuraman *et al*., 2020).

When the pathogen invades the host, the body produces large amounts of immunoglobulin (Ig) by the immune system and releases them into the blood stream, including IgG, IgM, and IgA. IgM is usually the first antibody produced in response to the virus invasion (Schroeder & Cavacini, 2010). IgG is a significant class of immunoglobulins found in the blood, comprising 75% of total serum immunoglobulins and has long-term immunity and immunological memory (Schroeder & Cavacini, 2010). ELISA-based IgM and IgG antibody tests have greater than 95% specificity for the diagnosis of COVID-19 (Sethuraman *et al*., 2020). Regarding SARS CoV-2, in their preprint article Huan *et al*. (2020) revealed that both IgM and IgA had early responses, while IgG showed up later, so they suggest IgA should be included in a serological test, which may provide higher diagnostic accuracy for COVID-19.

Based on current data, the WHO (2020e) does not recommend the use of antibody-detecting rapid diagnostic tests for patient care but encourages the continuation of studies to establish their usefulness in disease surveillance and epidemiologic research. POC test analytical errors have been described as relatively common, and might impair patient care (Meier & Jones, 2005). Table 04 shows a comparison among the current methods available to detected COVID-19.

**Table 4:** Comparison of main methods available in Brazil to detect COVID-19.

Technology	RT-PCR	ELISA	CLIA	Immunochromatography
Molecule tested	Viral RNA	Antibodies -IgA/IgM and IgG	Antibodies -IgA/IgM and IgG	Antibodies -IgM and IgG Few detect viral antigens
Type of sample required	Nasopharyngeal swabs or other upper respiratory tract specimens	Serum or plasma	Serum or plasma	Blood, plasma or Serum
Laboratory or point of care	Laboratory based	Laboratory based	Laboratory based	Point of care
Time to results	3-4 hours	1-5 hours	1-5 hours	15-30 minutes
Advantages	Gold Standard Multiple samples at once Acute phase detection	Multiple samples at once Identification of previous infection	Multiple samples at once Identification of previous infection	Fast
Disadvantages	Needs trained personnel Assays are easy to contaminate Difficult collection of sample	Needs trained personnel Detection limit for asymptomatic individuals	Needs trained personnel Detection limit for asymptomatic individuals	High rate of false negative

## 5. Procedures in IVF laboratory - general recommendations

In the IVF universe, there are recommendations and protocols for infection control and laboratory safety (Steyaert *et al*., 2000). There is no scientific evidence of COVID-19 transmission via gametes and embryos (Cochrane Gynecology and Fertility, 2020), but it is necessary to adopt universal standard precautions when handling samples (PRONUCLEO, 2020). We summarize below some recommendations regarding general procedures in the IVF laboratory:

- Laboratories should have the minimum number of personnel required to perform all quality control activities to ensure gamete safety and performance of urgent cases (ASRM, 2020). It is recommended to divide a team into mini teams as a review routine (ESHRE, 2020).
- To restrict access for the accompanying person(s) (ESHRE, 2020).
- Embryologists who have returned to their activities for at least 14 days should take their temperature daily; those who have a temperature above 37.3ºC and present any symptom related to COVID-19 must report to the clinic’s human resources department and remain socially distant (PRONUCLEO, 2020).
- Embryologists should wear the necessary PPE, gloves, cap, and surgical mask. For procedures that generate aerosols (on the oocyte retrieval procedure), N95 mask, surgical mask, and facial shield or protection glasses (PRONUCLEO, 2020).
- For the andrologist, the necessary PPE are gloves, cap, surgical mask, face shield, or glasses in specific activities that generate droplets and aerosol (on the seminal processing) and use long-sleeved aprons (PRONUCLEO, 2020).
- Review of emergency action plans, if an embryologist and andrologist are unable to perform an activity, qualified personnel (De Santis et al., 2020) should replace them.
- Other employees, such as doctors or nurses, should be trained on the procedures for refilling liquid nitrogen in storage tanks (De Santis *et al*., 2020).
- Cleaning routine at the end of each procedure with disinfectant agents, following the manual of Good Laboratory Practices (GLP), with products certified as to their effectiveness and tests recommended in the Standard Operating Procedure (ESHRE, 2020). Disinfectants with solutions based on quaternary ammonium, sodium hypochlorite or hydrogen peroxide are recommended (ASEBIR, 2020a).
- All the necessary products for the laboratory use, after being received, must be disinfected with alcohol 70%, await evaporation of the product and stored in its appropriate location (PRONUCLEO, 2020). Strict hygiene standards must be observed for all professionals, including aseptic techniques (ASEBIR, 2020a).
- The laboratory must be subjected to maximum air filtration, through HEPA filters (high-efficiency particulate air) and control Volatile Organic Compounds (VOC) (Meseguer *et al*., 2020; ESHRE Guideline Group on Good Practice in IVF Labs, 2016).
- All bodily fluids must be considered infectious (follicular fluid, blood, semen, etc.). Their manipulation must be quick and effective. Handled on the Class II safety booth or vertical laminar flow using N95 masks to prevent aerosol contamination. (ASEBIR, 2020a).

### 5.1 Recommendations for oocyte retrieval

ESHRE has made some recommendations for oocyte retrieval based on triage results (Table 05), and it provides an ART triage questionnaire that can be used/adapted for the triage of both staff and patients. In cases of positive screening and positive SARS-CoV-2 tests, oncologic patients and those who are at high risk of ovarian hyperstimulation syndrome must continue the treatment, and measures should be adopted to reduce risks of transmission to staff members (ESHRE, 2020).

**Table 5:** Possible scenarios for oocyte retrieval during the COVID-19 pandemic according to ESHRE.

Possible Scenarios	Recommendation
Scenario I (Include)	Standard procedures should be followed unless changes occur between ovulation trigger and oocyte retrieval
Scenario II (Case dependent)	If positive re-triage, consider testing for COVID-19. Based on the result, decide whether to continue the treatment or to postpone it
Scenario III (Exclude)	If the patient test positive for SARS-COV-2/COVID-19, before ovulation trigger or embryo thawing, postpone treatment, refer and isolate.

It is essential to mention that other measures should be adopted to reduce the risks of transmission to staff members, as follows:

- The patient must wear a surgical mask, being removed only during the anesthetic process (PRONUCLEO, 2020).
- All the teams must wear PPEs (ESHRE, 2020).
- Disinfection of operating theatre after the procedure (ESHRE, 2020).
- Follicular fluid must be handled in a laminar flow cabinet (ASEBIR, 2020a). If a laminar flow cabinet is not available, it is recommended to wear PPEs such as goggles and N95 masks (ASEBIR, 2020b).

### 5.2 Recommendations for andrology

PRONUCLEO has advised that the embryologist of the andrology laboratory should adopt the use of universal standard precautions when handling samples. They suggest following GLP as recommended by the WHO and by the Centers for Disease Control and Prevention Center (CDC), which includes training all personnel in the use of PPE (PRONUCLEO, 2020).

The collection room must be equipped with disinfection material, such as water, neutral soap, and 70% alcohol gel (PRONUCLEO, 2020), and the laboratory room must be disinfected after the procedure with disinfectant agents (Meseguer *et al*., 2020). The patient must be instructed on hand hygiene also on the genital organ before the collection procedure, after the collection, also perform hand hygiene procedures (PRONUCLEO, 2020).

### 5.3 Recommendations for embryo transfer

ASRM has advised in the pandemic period the cancellation of all embryo transfers, whether fresh or frozen (ASRM, 2020). ESHRE has suggested performing transfers only in low-risk/asymptomatic patients and partners and applying a freeze-all policy for all patients and partners who became symptomatic after the oocyte retrieval (ESHRE, 2020). In Brazil, an embryo transfer schedule should be carried out according to updated recommendations from class entities (SBRA, REDLARA, SBRH, and PRONUCLEO) and ANVISA’s current regulations. The number of embryos to be transferred should be defined previously in a virtual manner to minimize the time of the patient/couple in the unit (PRONUCLEO, 2020). It is essential to mention that the patient must go to the procedure room wearing a surgical mask (PRONUCLEO, 2020).

### 5.4 Recommendations for cryopreservation

Currently, there is no proven evidence of the presence of infectious agents (viruses) in diseases transmissible to cryopreserved gametes or embryos of patients infected in culture medium or liquid nitrogen (Cobo *et al*., 2012). Another factor to consider is that the cryopreservation technique for the sample is diluted at each step of the procedure. It is estimated that the dilution for the vitrification and devitrification procedures is 1: 6.6 trillion (Cohen & Berger, 2020).

Still, the possibility of cross-contamination of a liquid nitrogen container cannot be ruled out (ASRM, 2020). Therefore, according to biosafety measures, consider the precaution for freezing procedures:

- The use of cryogenic gloves and masks as PPE for embryologists (ASEBIR, 2020a).
- The possibility of using sample storage tanks in the nitrogen vapor phase to freeze positive samples for the SARS-CoV-2 virus (ESHRE, 2020). High-security straws and vapor phase storage tanks should be used for cryopreservation of samples from COVID-19 positive patients.
- For patients who tested positive for SARS-CoV-2, their samples should be kept in storage tanks separate from the tanks used in the laboratory routine (PRONUCLEO,2020).

## 6. Conclusion

All topics presented in this review were prepared based on all current scientific knowledge aiming to minimize the risks of SARS-CoV-2 transmission as much as possible. In summary, the resumption of activities at IVF laboratories and clinics in Brazil depends on the strict incorporation of all protection measures by the staff and patients. There is still little information regarding the impact of SARS-CoV-2 on fertility, pregnancy, and IVF, but treatments must be carried out safely as we know asymptomatic people transmit the virus.
